# Multiplexed fiber meta-tip–based circular polarimetry for label-free pathological analysis of ischemic stroke

**DOI:** 10.1117/1.NPh.12.1.015012

**Published:** 2025-03-05

**Authors:** Wenlin Luan, Qingcheng Song, Quancheng Cheng, Chunhua Chen, Xia Yu

**Affiliations:** a Beihang University, School of Instrumentation and Optoelectronic Engineering, Beijing, China; b Beihang University, Hangzhou International Innovation Institute, Hangzhou, China; c Peking University Health Science Center, School of Basic Medical Sciences, Department of Human Anatomy and Histology and Embryology, Beijing, China

**Keywords:** circular polarimetry, optical fiber meta-tip, ischemic stroke, label-free

## Abstract

**Significance:**

We present an optical technology for a full-process label-free method for brain slice screening. This proposed multiplexed circular polarimetric method has the advantages of simple operation and high accuracy which may provide easily accessible evidence for the future diagnosis of related diseases.

**Aim:**

One of its missions is to provide a quantifiable, reproducible analysis methodology that can replace or supplement traditional qualitative, subjective pathological analysis.

**Approach:**

A label-free, sensitive, and rapid circular polarimetric method based on a multiplexed optical fiber meta-tip is proposed for the digital pathology of ischemic stroke. Polarization information of forward-scattered light is used to identify pathological variations of axon distribution in ischemic stroke tissues. The newly designed optical fiber meta-tip with four channels offers miniature illumination in the multiplexed circular polarimetry method.

**Results:**

Our automated approach achieves more than 90% area under the curve in classifying ischemic stroke brain tissue in around 1 min.

**Conclusions:**

The high-sensitivity and label-free circular polarimetric method based on the multiplexed optical fiber meta-tip renders its potential for rapid digital pathology of various diseases. It will empower the application of digital pathology in future disease diagnosis by quantitatively introducing a new reliable data modality without altering currently established processes.

## 1 Introduction

**Fig. 1:**
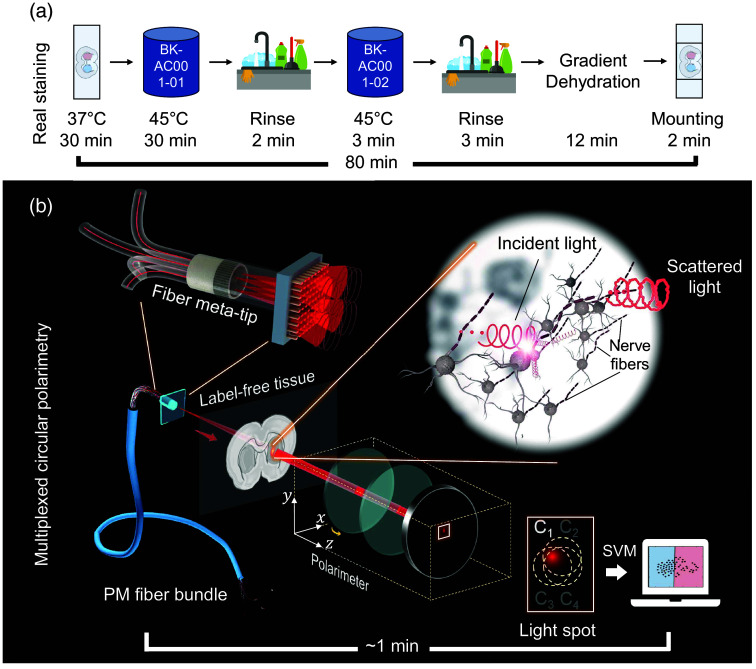
Schematic diagram of the (a) real myelin staining procedure of rat brain sections and (b) multiplexing circular polarimetric method based on a four-channel optical fiber meta-tip.

Neurodegenerative disorders (NDDs) are a set of heterogeneous disorders increasing in prevalence, especially in aging societies.^1^ Among NDDs, stroke is the second leading cause of death and a major cause of disability worldwide.^2^ In particular, ischemic stroke accounts for over 80% of all stroke cases.^3^ Due to the high incidence and mortality of ischemic stroke and its complex etiology, pathologists have constructed a variety of animal models of cerebral ischemia to simulate human stroke. Research efforts are aimed at discovering more effective drugs for stroke prevention and treatment, focusing on aspects of pathology, neurobiology, and pharmacology.^4^ The pathological analysis of stroke mainly uses the 2,3,5-triphenyl tetrazolium chloride method, where the area of viable cells and ischemic damage are marked in different colors. The damaged brain tissue can be calculated through image analysis to determine volume. However, for neurobiologists, other chemical or immunostaining methods are preferred, such as myelin staining, when studying damage and repair processes. These real staining methods can provide intuitive evidence for diagnosis; however, they are time-consuming, labor-intensive, and destructive to tissues. In addition, staining experiments are greatly influenced by experimenters and experimental environments. Digital pathology aims at digitizing images captured under bright-field microscopes. This innovation facilitated convenient storage and transmission, as well as enabled unbiased, quantitative, and reproducible diagnostic analysis. However, the reliance on labor-intensive and time-consuming tissue staining procedures persists, which limits the automation and speed of digital pathology workflows. Taking myelin staining of nerve fibers as an example, as shown in Fig. 1(a), the staining process takes up to 80 min. The ideal techniques would deliver rapid and accurate quantitative evidence to assist in decision-making. Therefore, label-free techniques have emerged as a key focus for the recent development of digital pathology.^5^

Is there any label-free imaging method that inherently provides high contrast about pathological information? Scattering in biological tissues, especially multiple scattering, alters the state of polarization (SOP) of light.^6^ In pathological studies related to fibrous structures or fibrotic processes, it is polarimetry that is preferred as a label-free method to increase the contrast of fibrous structures in images. The principle of polarimetry increasing contrast is based on the fact that fibrous structures modeled by optically uniaxial crystal^7^ have different effects on the SOP of scattered light compared with the surrounding biological tissue. In 2017, Qi et al.^8^ applied polarimetry to imaging of liver and breast tissue. The measured two metrics, depolarization and retardance, are then used to enhance the contrast of fiber components (e.g., central lobules and breast ducts) in the original image. In 2023, they combined the polarimeter with an ordinary endoscope to improve the accuracy of detecting laryngeal cancer. The test is based on the fact that cancerous cells destroy the original epithelial tissue by retarding the fibrils such as the lining of the larynx surface as well as the duct of glands in the larynx. Compared with judging by the hue value and saturation of ordinary color images, using depolarization and retardance of polarization detection increases the accuracy from 85% to 92% with the same image processing method.^9^ The abundant axons in brain tissue also act as optically uniaxial crystals.^7^ Recently, there have been a few studies using polarimetry methods for the diagnosis of NDDs in brain tissue. In 2021, Borovkova et al.^10^ conducted polarimetric measurements on a mouse model to screen for Alzheimer’s disease. The SOP of backward-scattered light spots is measured, resulting in a statistically significant differentiation degree of polarization (DOP) between mild and severe Alzheimer’s in paraffin-embedded mouse brain sections. However, further classification is not conducted.

In this work, we aim to develop a label-free and sensitive technique for tissue pathological analysis based on polarimetry. We propose a multiplexed circular polarimetry method. The measured polarization metrics inform us of the degree of injury and the calculated statistical moments of them offer spatial texture information of tissue on the normal side from that on the ischemic stroke sides. When tested on frozen brain sections of ischemic stroke model rats, the multiplexed circular polarimetry achieves >90% accuracy for classifying the normal and ischemic sides of the tissue. To better understand the circular polarimetry differentiation principle of ischemic stroke on brain tissue, we applied a Monte Carlo (MC) framework^11^^–^^13^ to simulate the main tissue structural changes caused by ischemic stroke. We find that the simulation results are in good agreement with the trends observed in the experiments. On the other hand, a compact optical design with multiplexing and switching functions is necessary in such a miniaturized modality. Metasurfaces emerging in recent years have revolutionized the design of optical devices and can solve challenging problems encountered by traditional optical devices^14^ which are capable of manipulating the incident light field on the subwavelength thickness. Optical fiber meta-tips composed of metasurface and optical fiber devices have the great advantages of compactness and flexibility, which have been applied in the biomedical field.^15^^–^^17^ We here designed a fast-switching multiplexed fiber meta-tip for high speed and distinct screening sensitivity and specificity. Compared with existing techniques for ischemic stroke pathological analysis, our multiplexed fiber meta-tip–based circular polarimetry has both efficiency and accuracy by calculating the distribution moment of polarization metrics that appear as a highlight at the ischemic area of tissue.

## 2 Methods and Materials

### 2.1 Brain Tissue Sample Preparation and Statistical Analysis

In this study, brain tissue samples are from six rats. Photos of these brain sections are shown in Fig. S1 in the Supplementary Material. Male Sprague–Dawley rats weighing 280 to 320 g, obtained from the Department of Laboratory Animal Science of Peking University Health Science Center, were used as the animal model in this study. The middle cerebral artery occlusion (MCAO) model was induced by occluding the middle cerebral artery (MCA) for 90 min using a silica gel suture (L3800, Jialing, Guangzhou, China).^18^ To occlude the origin of the MCA, a suture with a diameter of 0.36 to 0.40 mm was gently inserted from the external carotid artery to the internal carotid artery. After 90 min, the suture was withdrawn to allow reperfusion of the MCA. The rats were housed under a 12-h light–dark cycle, with *ad libitum* access to food and water.

Dihydromyricetin (DHM) has been proven as an effective drug for ischemic stroke.^19^ The treatment protocol for the DHM-MCAO group involved the administration of $100\;\mathrm{mg}\cdot {\mathrm{kg}}^{-1}$ DHM (SD8280, Solarbio, Beijing, China) for each injection. When reperfusion was initiated, the HP-β-CD (HP-β-CD ((2-hydroxypropyl)-β-cyclodextrin) solution ($4\;\mathrm{mL}\cdot {\mathrm{kg}}^{-1}$, 848 mg HP-β-CD dissolved in 4-mL normal saline) was intraperitoneally injected into rats randomly selected from the MCAO groups as the vehicle control. DHM solution ($100\;\mathrm{mg}\cdot {\mathrm{kg}}^{-1}$, 100 mg DHM dissolved in 40-mL HP-β-CD solution) was intraperitoneally injected into treated rats. The injections were given once daily for 7 days. After 7 days of injections, samples were taken. The brains were sectioned using a sliding vibratome (CM3050S, Leica, Buffalo Grove, Illinois, United States) to obtain coronal cryostat sections (20  μm thick). For the myelin staining of the brain sections, high resolution and contrast myelin staining was achieved using the gold phosphate derivative, TrueGold Kit (BK-AC001, Oasis Biofarm Inc., Hangzhou, China), following published protocols.^20^ It should be noted that the tissue staining here is only for result analysis after label-free measurement and has no impact on the measurement process. Myelinated axons are observed as red fibers throughout the brain tissue due to TrueGold staining. The brightfield photos taken with a light microscope are shown in Fig. S2 in the Supplementary Material. It can be observed that the morphology of myelinated axons is significantly different after the ischemic stroke occurs. Ethical approval for all animal experiments was obtained from the Animal Research Welfare Committee of Peking University Health Science Center.

Statistics and reproducibility data are expressed as mean ± standard deviation (SD) as indicated in each figure legend. A two-tailed Mann–Whitney test obtained the statistical significance between the two groups to assess the statistical significance of the differences, with P values<0.05 considered significant.

### 2.2 Circular Polarimetry for Pathological Analysis of Ischemic Stroke

**Fig. 2:**
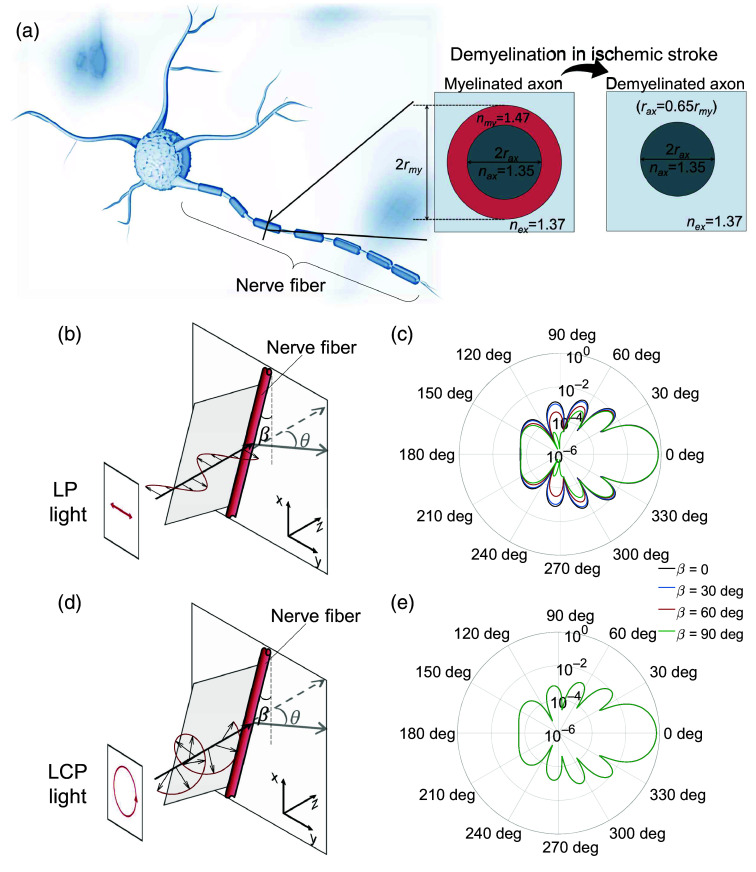
Monte Carlo simulation of ischemic stroke. (a) Cylinder scatterers in the model mimic the nerve fibers. Scattering patterns of the (b) and (c) linearly and (d) and (e) circularly polarized illumination.

The polarization characterization of brain tissue mostly depends on the distribution of the fiber-like axons inside and reflects on the polarization state of scattered light.^21^ To simulate the pathological changes in ischemic stroke, we employ MC simulations, where axons are modeled as cylindrical scatterers embedded in a homogeneous surrounding medium as shown in Fig. 2(a). It is found that the total density of axons in ischemic brain tissue remains the same, and the density of unmyelinated axons is 50 times that of myelinated axons.^3^ The direction of axonal rotation becomes more chaotic. These pathological changes in ischemic stroke are modeled by adjusting the density, diameter, refractive index, and orientations of the axons.^22^^,^^23^ More details are available in Supplementary Material.

Polarization states of the illumination light affect the detection sensitivity. Figures 2(b)–2(e) show that scattering patterns from cylindrical scatterers differ under linearly polarized light, whereas they are consistent for circularly polarized light. As axonal orientation in brain diseases is unknown, circularly polarized light is preferred for ischemic stroke detection because it is independent of fiber orientation. In general, the circular polarimetric method measures the full Stokes vector $\vec{S}=(S_{0},S_{1},S_{2},S_{3}{)}^{T}$ of light and obtains depolarization (dep) and retardance (δ). The calculation of these two polarization metrics is shown in Eq. (1), where the DOP and $\hat{s}$ can be obtained directly from the Stokes vector, as shown in Eq. (2)

dep=1−DOPoutDOPin,δ=cos−1 (s^in·s^out|s^in||s^out|), (1)

DOP=S12+S22+S32S0,s^=(S1,S2,S3)T. (2)

Finally, the MC simulation modeled the response of normal brain tissue and ischemic stroke-damaged brain tissue to circularly polarized incident light and calculated the polarization metrics of forward-scattered light: depolarization (dep) and retardance (δ). Results show that depolarization of forward-scattered light increases from 0.14 to 0.35, and retardance increases from 0.25 to 0.33, highlighting the potential of these polarization metrics (dep and δ) in distinguishing ischemic brain tissue from normal tissue.

**Fig. 3:**
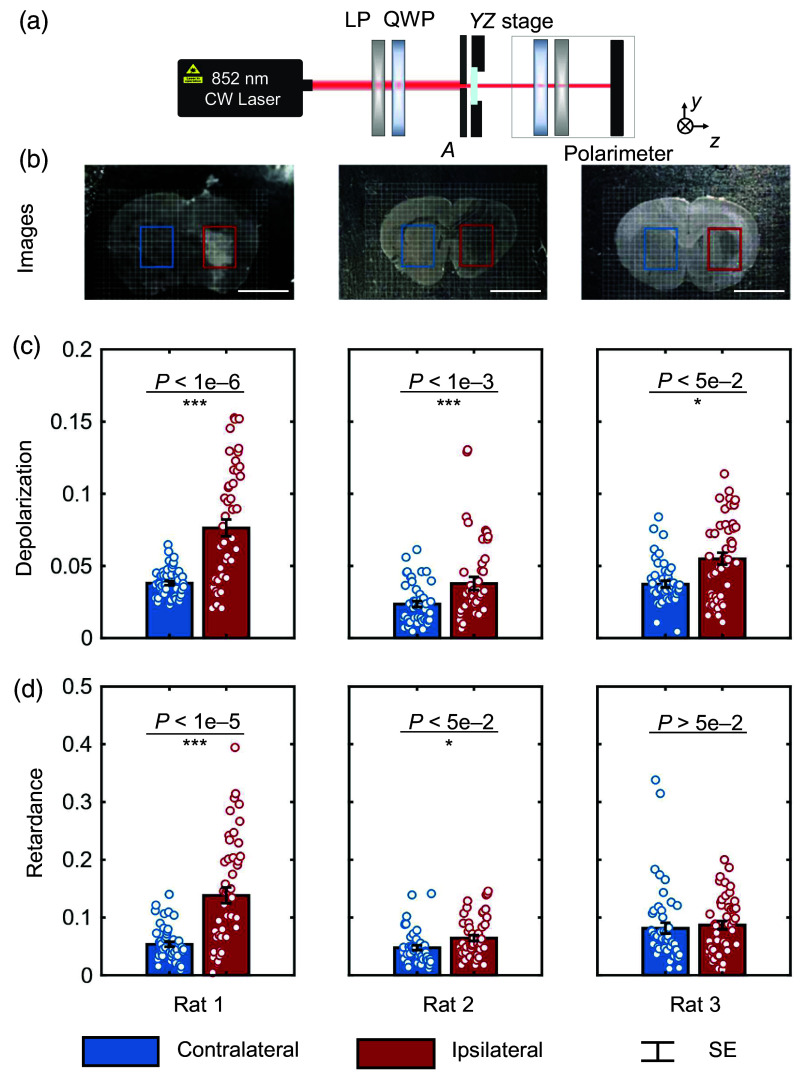
Experiment and samples of circular polarimetric measurement on the brain sections from rats 1 to 3. (a) Experimental setup of circular polarimetric measurement. (b) Pictures of unilateral ischemic stroke samples from rats 1 to 3. The scale bar is 5 mm. The values of (c) depolarization and (d) retardance of the detected light from the contralateral and ipsilateral areas of tissues in panel (b). Data are mean ± SD. *P<0.05; **P<0.01; ***P<0.001.

Preliminary experiments are shown to have the same trend as the simulation results. To experimentally explore the influence of ischemic stroke on the polarization of forward-scattered light, circular polarimetric measurements were conducted on frozen sections of rats’ brains, and two polarization metrics, depolarization dep and retardance δ, were calculated for characterization. The experimental system is shown schematically in Fig. 3(a).

The light source is a semiconductor laser with a center wavelength of 852 nm. The emitted light is transmitted into circular polarization by passing a linear polarizer and a quarter-wave plate. The circularly polarized beam is then reduced after an aperture (A) with a diameter of ∼500  μm and illuminates the tissue. It is worth noting that 500  μm is a half-empirical value suitable for rat brains. As shown in Fig. S2(c) in the Supplementary Material, morphological analysis was performed using 500×500  μm as a unit on bright-field micrographs of myelin-stained brain tissue, resulting in significant differences in optical density of stained myelinated axons. It can be adjusted to adapt to different organisms and organs. The sample is fixed on a two-dimensional translation stage that allows spatial scanning controlled by the computer. The measured region is located in a 3×4  mm area in rats’ striatum, and 48 sampling points are obtained by a two-dimensional zig-zag scanning with a step of 500  μm from each side of MCAO rats’ brain. The forward-scattered light from the sample is collected and directed to the Stokes polarimeter. The polarimeter performs polarization measurements based on the rotating quarter-wave plate method. Specifically, the intensity is measured every 15 deg as the wave plate rotates from 0 to 180 deg, and the polarization data Stokes vector is obtained through Fourier analysis. The samples in the current measurement were three frozen sections containing unstained fixed brain tissue from three unilateral MCAO rats. Among them, rat 3 received DHM treatment after MCAO surgery, whereas rats 1 and 2 did not receive drug treatment. In each brain section, the ipsilateral side of the hemisphere that underwent MCAO surgery was used as the ischemic brain tissue sample, whereas the contralateral side was used as the normal brain tissue sample. The unstained brain tissue sections from three unilateral MCAO rats utilized in the study are shown in Fig. 3(b).

The circular polarimetric measurement results in Figs. 3(c) and 3(d) have shown that the polarization metrics of light forward-scattered from the contralateral and ipsilateral areas of rats’ brains are significantly different in most cases. The results manifest that the occurrence of ischemic stroke had a significant impact on both considered polarization metrics. As Fig. 3(d) shows, the ipsilateral area presented an increase in depolarization compared with its symmetrical contralateral area. The same tendency was observed in retardance in Fig. 3(e). One possible reason for this change is that the ischemic tissue scatterers light more complexly, resulting in a longer optical path within the tissue. This phenomenon could potentially account for the observed elevation in depolarization and retardance within ischemic tissue. Notably, although the significance of the difference in the two polarization metrics decreases for rat 3 from the DHM-MCAO group compared with the front two rats from the MCAO group, the P value of depolarization still suggests a statistically significant difference (P<0.05). This demonstrates that circular polarimetric measurements can reflect different degrees of injury. Furthermore, even when the degree of injury is mild (or after a certain degree of treatment), it still has a statistically significant impact on the polarization state of light. The results primarily demonstrate the capability of the circular polarization method in digital pathology.

### 2.3 Four-Channel Circular Polarimetry for Pathological Analysis of Ischemic Stroke

High sensitivity and specificity in classifying the normal and damaged tissue are necessary for ischemic stroke pathological analysis. Tissue spatial texture also contains valuable information for diagnosis. Previous work performed statistical moment operations on the pixels of polarization imaging images and used them to classify different types of tissues.^7^ Similarly, the proposed four-channel method here extracts spatial distribution information by measuring the polarization characteristics of tissue at four adjacent spatial locations, thereby improving the accuracy of pathological analysis. $Z_{1}$ to $Z_{4}$ are calculated as Eqs. (3)–(6), representing the mean value, standard deviation, skewness, and kurtosis. Here, $C_{j}$ stands for the metrics measured by the j’th channel.

$$Z_{1}=\frac{1}{4}\overset{4}{\underset{j=1}{\sum }}C_{j},$$ (3)

$$Z_{2}=\sqrt{\frac{1}{3}\overset{4}{\underset{j=1}{\sum }}(C_{j}-Z_{1}{)}^{2}},$$ (4)

$$Z_{3}=\frac{1}{Z_{2}^{3}}\frac{1}{4}\overset{4}{\underset{j=1}{\sum }}{\left(C_{j}-Z_{1}\right)}^{3},$$ (5)

$$Z_{4}=\frac{1}{Z_{2}^{4}}\frac{1}{4}\sum_{j=1}^{4}(C_{j}-Z_{1}{)}^{4}.$$ (6)

**Fig. 4:**
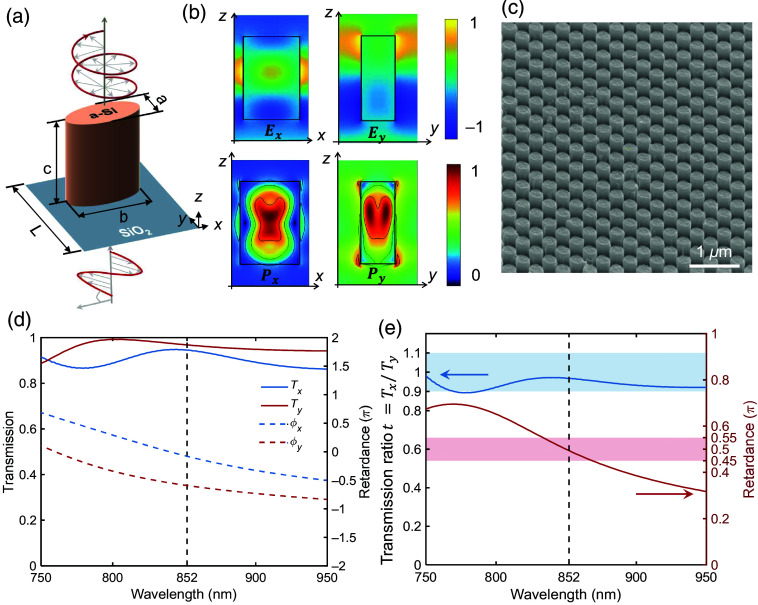
a-Si metasurface. (a) Schematic diagram of a single unit cell of the metasurface. (b) Simulated field distribution in the designed metasurface. (c) Scanning electron microscope photo of the fabricated metasurface. (d) Simulated transmission and retardance of elliptic nanopillar under x- and y-polarized incidence. (e) Simulated ratio of transmission ($t=T_{x}/T_{y}$) and phase difference of metasurface. Blue and red regions show the range of 0.9<T<1.1 and 0.45π<Δϕ<0.55π.

To rapidly and easily conduct the four-channel circular polarimetric measurement, a multiplexed optical fiber meta-tip is designed. Our optical fiber meta-tip is composed of a polarization-maintaining (PM) fiber bundle and an all-dielectric metasurface. The PM fibers produce linearly polarized light that is incident on the metasurface. The metasurface is designed as a linear-to-circular polarization converter. The angle between the slow axis of PM fibers and that of metasurface is 45 deg. Figure 4(a) depicts the schematic configuration of a single unit cell in the metasurface. The geometry of the unit cell is an elliptical cylinder, and the phase delay introduced by the metasurface can be controlled by rationally designing its geometric size. It was uniformly designed with a=130  nm, b=230  nm, and c=388  nm and arranged into a square lattice with period L=300  nm. Simulations of the metasurface were performed using the commercially available finite-difference time-domain method. As shown in the first column of Fig. 4(b), the x- and y-polarized electric fields experience different phase shifts after penetrating unit cells with periodic boundaries. The difference among them is exactly π/2 at 852 nm, where the main components in biological tissues have weak absorption.^24^ The map of Poynting vector $P_{x}$ and $P_{y}$ indicates that the energy of both polarization states is trapped in the dielectric column, which means the coupling among units is weak. Consequently, the simulation of the whole area covered by the array shown in Fig. 4(c) can be performed under the periodic boundary condition. The simulation results are shown in Figs. 4(d) and 4(e) for a normally incident x- and y-polarized plane wave, where the transmission T and phase retardance are plotted in Fig. 4(d).

Figure 4(e) depicts the ratio of transmittance ($T=\frac{Tx}{Ty}$) and the phase difference ($\Delta \phi =\phi_{x}-\phi_{y}$) of the metasurface. For an efficient quarter-wave plate, its bandwidth is typically defined as the phase difference (Δϕ) satisfying 0.45π<Δϕ<0.55π, as well as the ratio of transmittance between 0.9 and 1.1.^25^ The simulation results suggest that the metasurface operates as a quarter-wave plate in the wavelength range from 833 to 868 nm. The weak absorption and high refractive index of a-Si at a wavelength of 850 nm ensure the high transmittance and feasibility of the metasurface. The metasurface was fabricated with electron beam lithography on a ${\mathrm{SiO}}_{2}$ substrate. The ellipticity is defined as the ratio of the minimum to the maximum value in the SOP, and the perfect circularly polarized light has an ellipticity of 1. For an efficient quarter-wave plate, the ellipticity of the transmitted circularly polarized light should be larger than 0.9.^26^ Here, the ellipticity of the four-channel output from the optical fiber meta-tip is respectively measured as 0.93, 0.93, 0.92, and 0.92. Detailed assembly and measurement methods of the fiber meta-tip can be found in Figs. S4–S6 in the Supplementary Material.

**Fig. 5:**
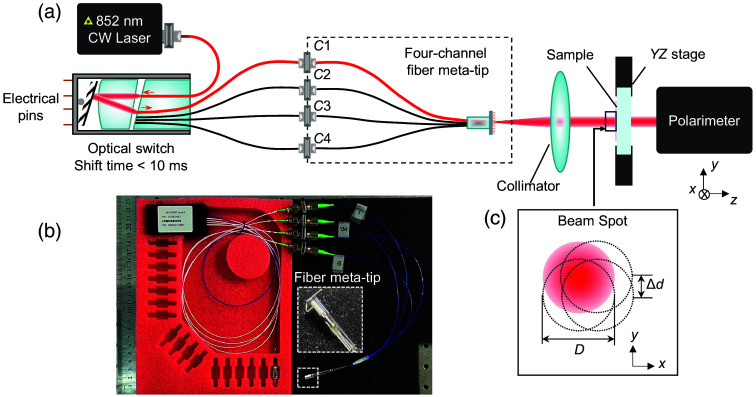
Schematic diagram of the multiplexed circular polarimetry method. (a) Experimental setup. Currently displays the measurement status by one channel [channel 1 (C1)]. (b) Picture of the attached four-channel fiber meta-tip and optical switch. The insert diagram shows the details of the fiber meta-tip. (c) Diagram of an illuminating spot on tissue. D∼500  μm, Δd=125  μm.

The experimental system is shown schematically in Fig. 5(a). The 852-nm wavelength laser source connects to a 1×4 PM optical switch (customized from E-PHOTICS). The optical switch realizes channels’ gating and switching through a computer-controlled microelectromechanical system. Its four output optical fibers connect to the four input PM fibers (PM-780HP) of the fiber meta-tip. Figure 5(a) currently displays the measurement status by one channel [channel 1 (C1)].

**Fig. 6:**
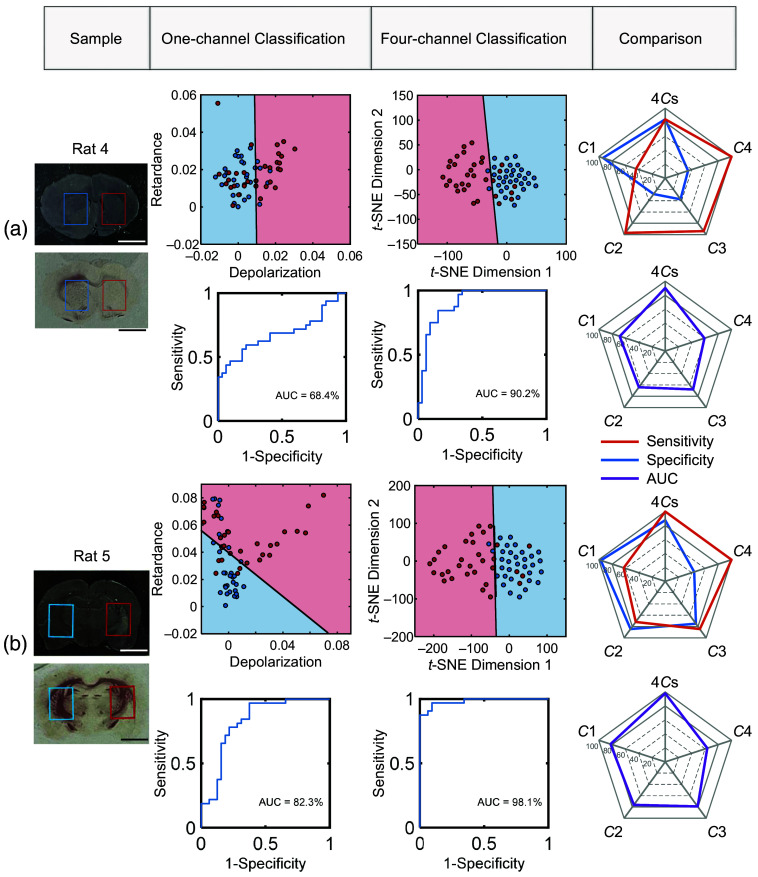
Classification results by one-channel and four-channel circular polarimetry. The scale bar is 5 mm. Comparison of the classification performance on samples (a) rat 4 and (b) rat 5 is based on two-dimensional scatter plots, ROC curves, the AUC, sensitivity, and specificity.

Picture of the attached four-channel fiber meta-tip and optical switch is shown in Fig. 5(b), and the insert shows details of the fiber meta-tip. The light passing through the fiber meta-tip is converted to be circularly polarized and collimated by a collimator. The collimator consists of a pair of lenses collimating the divergent light from the fiber into a beam of approximately D=500  μm. The collimated light spot is shown in Fig. 5(c), with the distance Δd=125  μm among adjacent spots. The four-channel circular polarimetric measurements were conducted on two unilateral MCAO rats. Rat 4 is from the DHM-MCAO group, and rat 5 is from the control group. The photos of the brain sections from these two rats are shown in the first column in Fig. 6. For each rat, 64 points were sampled from the symmetric regions of the brain. At each point, data from four channels were collected, including the depolarization (dep) and polarization angle (δ) for each channel, along with the first to fourth-order statistical moments of these two polarization metrics. The flow chart of the four-channel circular polarimetry is shown in Fig. S8 in the Supplementary Material, indicating the complete process from optical measurement to data analysis.

## 3 Results and Discussion

The classification results of single-channel and four-channel circular polarimetric methods are specifically shown in the second and third columns in Fig. 6. The data collected from the contralateral side are labeled as negative (normal), whereas the data from the ipsilateral side are labeled as positive (abnormal). Linear kernel support vector machine is used to perform classification, and the performance is evaluated through the calculation of the receiver operating characteristic (ROC) curve and the area under the curve (AUC). The sensitivity and specificity values are the true-positive rate and the complement of the false-positive rate when the maximum of (Sensitivity+Specificity)/2 is reached.^9^

To facilitate a more intuitive comparison of the separability between one-channel and four-channel methods, the four-channel data are reduced in dimensionality using t-distributed Stochastic Neighbor Embedding, as shown in Fig. 6. The results demonstrate that the four-channel method obtains better classification performance. As shown in Fig. 6, although the data from the contralateral and ipsilateral areas of tissue exhibit different distributions in the classification results using the one-channel circular polarimetric method, there is an overlap between two sets of data, and their adjacent data overlap. The ROC curves also demonstrate the imperfect classification, with 68.4% AUC for Rat 4 and 82.3% AUC for Rat 5. The scatter plot and ROC curve of one-channel measurement displayed in Fig. 6 are based on data from Channel 1 (C1). The ROC curves of all four-channel measurements on rats 4 and 5 are available in Fig. S7 in the Supplementary Material. The third column in Fig. 6 shows that the four-channel method can separate the two sets of data into two sets more distinctly, 90.2% AUC for rat 4 and 98.1% AUC for rat 5. In the fourth column, the classification performance by different methods is characterized and compared by sensitivity, specificity, and AUC. C1, C2, C3, and C4 refer to the classification based on polarization metrics measured by the first channel (C1) to the fourth channel (C4) of multiplexed optical fiber meta-tip; 4Cs refer to the classification results of four-channel circular polarimetry measured by the multiplexed optical fiber meta-tip. Using a commercial polarimeter (400  sampling/s) and a polarization-maintaining optical switch, this label-free method can measure and classify 48 points in ∼1  min, covering an area of 3×4  mm (see Video 1, MP4, 14.6 MB [URL: https://doi.org/10.1117/1.NPh.12.1.015012.s1]).

**Fig. 7:**
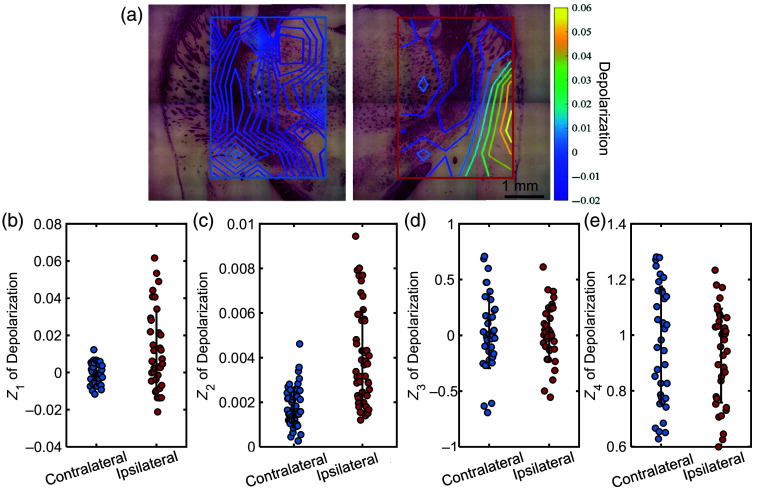
Result analysis. (a) Maps of depolarization from the contralateral (left) and ipsilateral areas (right) are superimposed on the corresponding positions of myelin staining sections’ micrographs. (b)–(e) Values of the first- to fourth-order statistical moments of depolarization from a tissue sample of rat 5.

In the results, we find that the statistical moments improved the classification performance of the four-channel circular polarization measurement. In the four-channel circular polarization measurement, after involving the statistical moments for classification, the AUC for rat 4 increases from 89.6% to 90.2%, and the AUC for rat 5 increases from 87.4% to 98.1%. After all the polarization measurements, the tissue sections are stained and photographed under a bright-field microscope for confirmation of our classification and further analysis of results. The contour plot of the polarization metrics dep is overlapped with the corresponding positions as shown in Fig. 7(a). Figures 7(b)–7(e) show the values of the first- to fourth-order moments of the depolarization. The mean value $Z_{1}$ in Fig. 7(b) reflects the overall polarization characteristics of the four-channel measurement area; the standard deviation $Z_{2}$ in Fig. 7(c) reflects whether the four-channel measurement data are concentrated or scattered, the skewness $Z_{3}$ in Fig. 7(d) reflects the concentration symmetry of the data among channels, and the kurtosis $Z_{4}$ in Fig. 7(e) reflects whether there is an abnormal value. The measurement of pathological tissue shows a larger mean and variance, whereas the skewness and kurtosis are similar to those of normal tissue. This indicates that the more severely damaged the tissue, the larger the dep value corresponding to the diseased tissue, and the data collected by the four channels on the ipsilateral side of the brain tissue are more dispersed. The statistical moment characteristic can be explained by the pathological characteristics of stroke. The brain tissue in ischemic stroke is characterized by a gradient distribution of injury, with the severity gradually decreasing from the ischemic core to the penumbra as shown intuitively in the afterward staining result of Fig. 7(a). This will be reflected in the spatial distribution of the measured polarization metrics, as shown in the contour map in Fig. 7(a).

In addition, the AUC of rat 4 is lower than that of rat 5. This may be the effect of drug treatment. DHM has been shown to effectively treat damage caused by ischemic stroke and reduce the area of injured tissue.^19^ Thus, the tissue on the ipsilateral side has partially or completely recovered. This may explain the observed decrease in classification accuracy of the rat brain after DHM treatment.

We note the following limitations currently hinder this approach from having true diagnostic applications: As the striatum is a selectively vulnerable area in the brain, it will be quickly damaged after ischemia and is one of the most sensitive brain regions to ischemia.^27^ Therefore, our research here focuses on the striatum. Although pathological processes such as demyelination occur similarly in both regions, we recognize that the denser distribution of axons in the cortex may present challenges for the current method. For example, the light penetration depth and resolution required for smaller nerve bundles could necessitate adjustments to the measurement parameters, such as optimizing the system’s sensitivity or reducing the spot size for cortical tissue. The interaction between light and tissue in the cortex could also differ due to the higher density of axonal fibers, which might affect the spatial texture extracted using statistical moments. These factors may require additional refinement for effective application in cortical tissue. On the other hand, if we want to move this technology to human tissue in the future, the spatial frequency of tissue pathology information will be different, which means that the spot size and channel spacing of the four-channel method need to be changed for optimal diagnosis. In addition, sampling and measuring 48 points in a 3×4  mm brain tissue area now takes ∼70  s. Moving the stage accounts for 96.8% of this time, with only 1.92 s spent on actual measurements. Reducing the number of sampling points can also decrease the time required without compromising classification accuracy. Therefore, it is believed that detection time can be shortened by optimizing the scanning method in future studies. This study also provides evidence for the application of a four-channel optical fiber meta-tip in multiplexed circular polarimetry. In the future, more diverse functions could be extended on top of the linear-to-circular polarization conversion, further enhancing device compactness or functionality.

## 4 Conclusion

Here, a label-free, automatic, and sensitive four-channel circular polarimetric method is proposed for pathological analysis of ischemic stroke. Sampling 48 points over a 3×4  mm brain tissue area takes ∼70  s. A new design of optical fiber meta-tip is realized for efficient generation of multi-channel circular polarization, achieving significant classification between normal and ischemic tissues, with >90% AUC. Our study also provides the first proof of concept of classifying ischemic stroke based on multiplexed circular polarimetry. To understand the underlying principle of how injury caused by ischemic stroke affects the polarization state of scattered light, a Monte Carlo simulation was conducted to simulate the changes in axonal structure and distribution. The simulation result showed the same trend as that of the experiment. The potential of the label-free, high-sensitivity, and rapid circular polarimetric method based on a multi-channel optical fiber meta-tip is evident in its applicability to digital pathology studies related to fibrous biological components or development. Moreover, this technology could be further explored in drug screening for related diseases, as a tool to greatly improve efficiency.

## Supplementary Material

10.1117/1.NPh.12.1.015012.s01

10.1117/1.NPh.12.1.015012.s1

## Data Availability

Original data and code underlying the results presented in this paper may be obtained from the authors upon reasonable request.
