# Supplementary material for: Multiplexed fiber meta-tip–based circular polarimetry for label-free pathological analysis of ischemic stroke
Source: Neurophotonics. 2025 Mar 5;12(1):015012. doi: 10.1117/1.NPh.12.1.015012 (PMC11888776; doi:10.1117/1.NPh.12.1.015012)
Supplement: Supplementary file 1 [file NPh_012_015012_SD001.pdf]

Multiplexed fiber meta-tip based circular polarimetry for label-free pathological analysis of ischemic stroke

Wenlin Luan,[a,b,†] Qingcheng Song,[a,†] Quancheng Cheng,[c] Chunhua Chen,[c] Xia Yu[a,b*]

[a]School of Instrumentation and Optoelectronic Engineering, Beihang University, Beijing 100191, China

[b] HangzhoSu International Innovation Institute, Beihang University, Hangzhou 311115, China

[c]Department of Human Anatomy and Histology and Embryology, School of Basic Medical Sciences, Peking University Health Science Center, Beijing 100191, China

SUPPLEMENTAL MATERIAL

A. Brain tissue samples

In this study, brain tissue samples are from six rats. Photos of these brain sections before and after staining are shown in Figure S1. Myelinated axons are observed as red fibers throughout the brain tissue due to TrueGold staining.

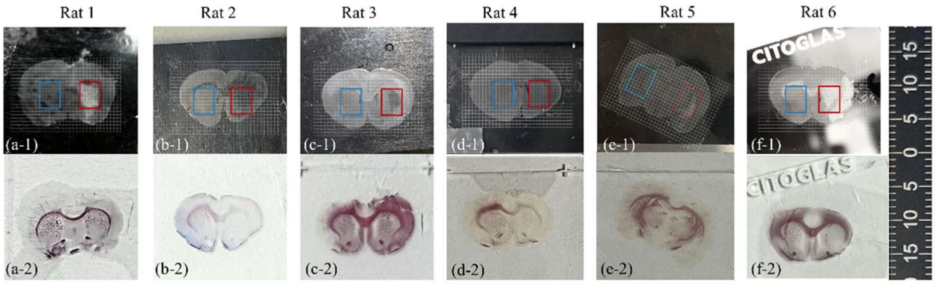

Fig. S1. Unilateral ischemic stroke sample involved in the experiment. (a-1)-(f-1) The encapsulated tissue samples captured under a black background. The samples are from Rat 1 to Rat 6. The red and blue regions represent the ischemic and normal white matter areas tested in the experiment. The side length of finer mesh grid is 500 μm. (a-2)-(f-2) Tissue samples after myelin staining.

The brightfield photos taken with a light microscope are shown in Figure. S2b,d. It can be observed that the morphology of myelinated axons is significantly different after the ischemic stroke occurs by the morphological difference and statistic result as shown in Figure S2c.

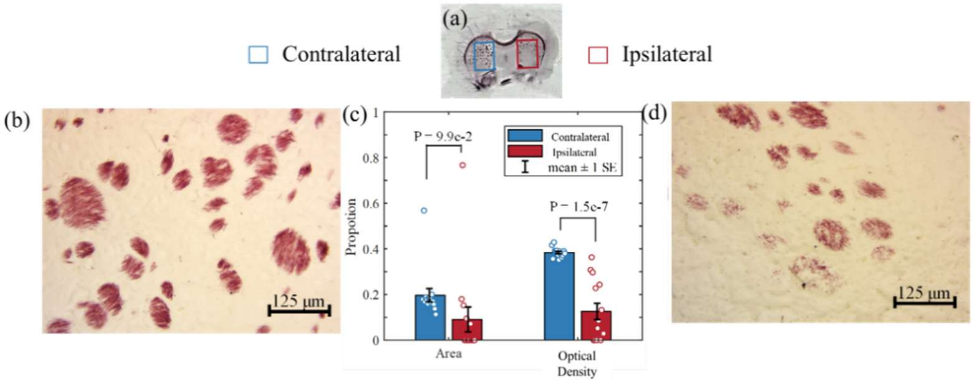

Fig. S2. (a) Photo of whole brain tissue from Rat 6 after myelin staining. (b) Contralateral and (d) ipsilateral side of bright-field photomicrographs taken by a light microscope. (c) Statistical result of morphological analysis performed with 500 µm × 500 µm as a unit on bright field micrographs of myelin-stained brain tissue. The Mann-Whitney test was used to assess the statistical significance of the differences and the P values are listed in the corresponding graphs.

B. Monte Carlo simulation

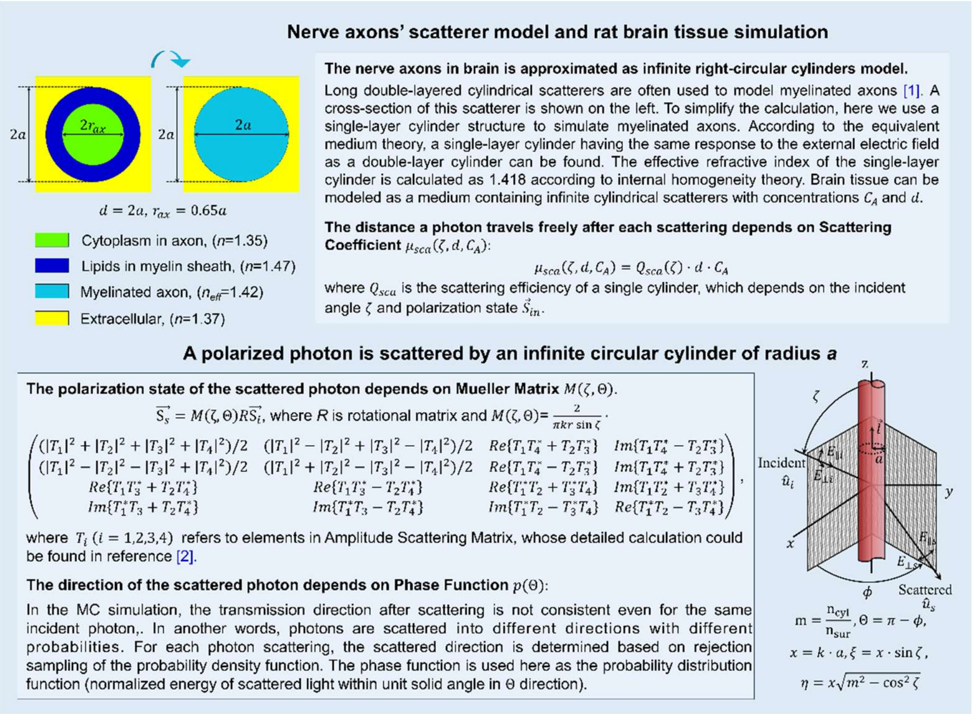

Fig. S3. In the Monte Carlo simulation, myelinated nerve axons and unmyelinated nerve axons are modeled as infinite long cylinders [21]. The SOP and direction of the photon can be calculated after a single scattering of the photon by the cylindrical axon.[23]

The simulation was implemented based on a framework similar to that in reference [23]. In this program, axons in brain tissue are simulated as cylindrical scatterers with radius a, refractive index n, and direction vector $\vec{l}$ in a homogeneous surrounding medium as shown in the first part of Figure S3.

Each photon in the simulation incidents along the direction vector $\overrightarrow{u_i}$. The single scattering process is shown in the second part of Figure S3. The polarization state of the scattered photon relies on the Mueller Matrix $M(\zeta, \Theta)$, which is related to the incidence angle ζ and azimuth angle Θ. Details of the scattering model can be found in reference [23] In MC simulation, Θ is determined by the rejection sampling method based on the phase function $p(\Theta)$ [11].

In the normal brain tissue simulation, the density of myelinated axons is set 10^6 cm^{-2} and the diameter d_m is 1.5 μm. 10^6 cm^{-2} is a typical magnitude of the axons' density in rats' brains [22]. The cylinders mostly lie on a plane perpendicular to the incident direction of light, with a variance of 10° [21]. Since the slice thickness is 20 μm, we mainly consider here that the angle between the cylinder axis and the plane does not exceed10°. Caused by demyelination during ischemic stroke, it is found that the total density of axons in ischemic brain tissue remains the same and the density of unmyelinated axons is 50 times that of myelinated axons [3]. And the direction of axonal rotation becomes more chaotic. Here, the axonal azimuth angle after ischemic stroke is set to a normal distribution with a variance of 45°. According to the myelinated axons' model in Figure S3, the diameter of the unmyelinated axons is set to 0.65 times that of the myelinated axons, and the refractive index is approximately that of the cytoplasm. The incident circularly polarized light is scattered from normal and ischemic brain tissue and collected to measure their polarization states.

C. Optical fiber meta-tip

Here, a two-step integration method was used to assemble the optical fiber meta-tip as shown in Figure S4. The first step was fabricating a multiplexed fiber bundle. The fiber bundle consists of four PM780-HP fibers with flat and parallel end faces and is embedded in a ceramic ferrule. The four polarization-maintaining optical fibers are clamped in V-grooves to ensure parallelism and be rotated to fast axis alignment with the assistance of a vision system. The second step involved precisely appending the metasurface to the fiber bundle. A glass tube (dimensions: 5 mm outer diameter, 3 mm inner diameter, and 30 mm height) was used to connect the metasurface and the fiber bundle. The fiber bundle was firmly fixed on a motorized translation stage, and its position within the glass tube was precisely adjusted to match that of the metasurface. UV-curable epoxy was used to bond the fiber bundle, glass tube, and metasurface together. The prepared four-channel fiber meta-tip is as Figure S5.

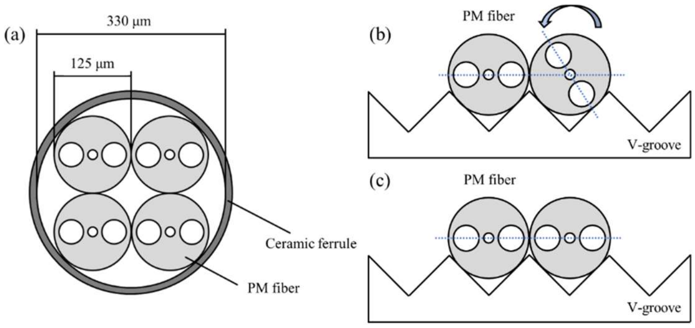

Fig. S4. Schematic of fiber meta-tip assembly. (a) Schematic representation of the cross-section of fiber bundle. Four PM fibers have their slow axes aligned in parallel. (b-c) Fabrication process of aligning two PM fiber to parallel. With the assistance of a V-groove, fiber was aligned and rotated within the visual system.

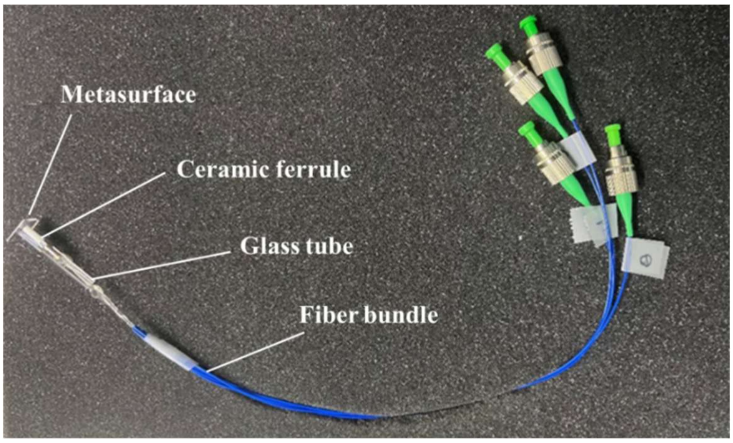

Fig. S5. The image of the four-channel fiber meta-tip.

Light passing through the metasurface from four channels passes through a linear polarizer (LP) and enters the power meter as shown in Figure S6. The maximum I_{max} and minimum I_{min} values of the polarizer during one rotation are recorded, and the ellipticity ϵ is calculated by $\epsilon = \sqrt{I_{max}/I_{min}}$.

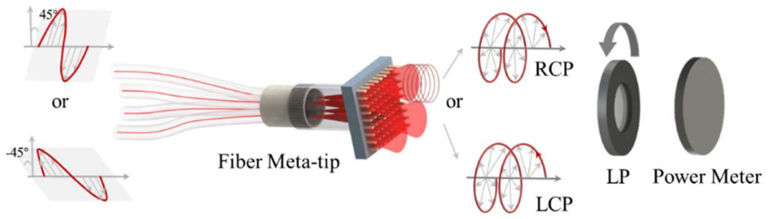

Fig. S6. The experimental setup used to measure the ellipticity of the output of fiber meta-tip.

D. ROC curves of the four channels of Rat 4,5 samples

The ROC curves of all four-channel measurements on Rat 4,5 are shown in Figure S7.

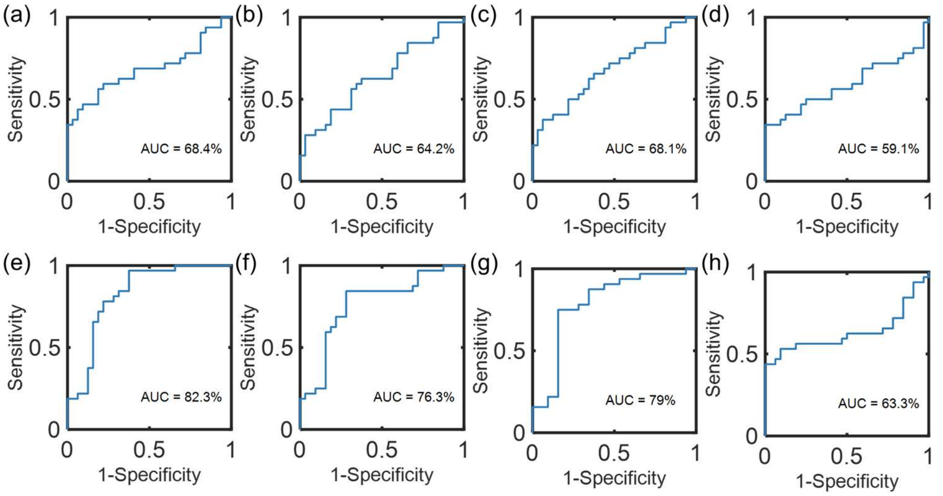

Fig. S7. The ROC curves of all four-channel measurements on (a-d) Rat 4 and (e-h) Rat 5.

E. Flow chart of measurement

The flow chart in Figure S8 indicates the complete process from optical measurement to data analysis.

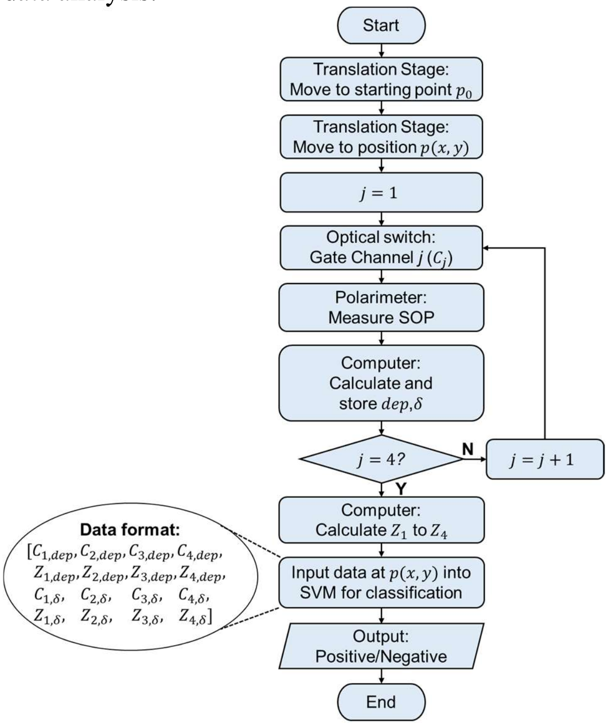

Fig. S8. Flow chart of the four-channel circular polarimetric measurement.
